# Supplementary material for: Caring in the silences: why physicians and surgeons do not discuss emergency care and treatment planning with their patients — an analysis of hospital-based ethnographic case studies in England
Source: BMJ Open. 2022 Mar 6;12(3):e046189. doi: 10.1136/bmjopen-2020-046189 (PMC8905976; doi:10.1136/bmjopen-2020-046189)
Supplement: Supplementary data [file bmjopen-2020-046189supp001.pdf]

ReSPECT Evaluation Study:
Semi-Structured Interview Topic Guide for Clinician Participants

Case-based questions:

1. Can you talk me through the process of completing the ReSPECT form for patient X?
    a. Or, I noticed that you didn't complete a ReSPECT form for patient Y. What were the reasons for that?
2. What did you take into account in making your recommendations for future treatment?
3. How did you feel about the conversation?
    a. Did you find anything about the conversation difficult?
4. What influenced your decision?
    a. Did you find anything about making the decision difficult?

Repeat the above questions for cases 2 and 3 (if relevant).

General questions:

1. When do you usually use the ReSPECT form?
    a. What circumstances make it more likely for you to complete the ReSPECT form?
    b. What circumstances make it less likely for you to complete the ReSPECT form?
    c. Under what circumstances do you update ReSPECT forms?
2. How do you decide when to hold the ReSPECT conversation?
    a. How do you time ReSPECT conversations in relation to your other activities?
    b. Do you gauge patients' emotional readiness for the ReSPECT conversation before starting the discussion? If so, how?
    c. Are there cases when the emotional burden of having a conversation outweighs the risk of not having a conversation?
3. How do you start a typical ReSPECT conversation?
    a. Where do you situate the CPR discussion within the ReSPECT conversation? How does that affect the rest of the conversation?
4. In many ReSPECT conversations, patients are asked to imagine a difficult future scenario. How do you present these future scenarios to your patients and their relatives?
    a. Do you sometimes doubt the ability of patients and relatives to imagine these potential scenarios? In these cases, what do you do?
    b. Should a patient who prefers not to know what their future holds be asked to imagine these scenarios?
5. What is the role of the patient's family in the ReSPECT conversation?
6. During the ReSPECT conversation, what approaches do you use to maintain rapport with patients? With patients' families?
    a. Does the ReSPECT form influence your discussions with patients and families?
    b. Do you think the ReSPECT conversation affects the building of trust between doctor and patient? If so, in what ways?
    c. How do you make sure that patients and families understand what has been discussed and decided in the ReSPECT conversation?
7. Thinking about the spaces and times in which you usually hold ReSPECT conversations, how do these settings shape the conversation?
    a. Do you feel that different ward environments influence patients' ability to engage with the ReSPECT conversation?

    b. Do you feel that these ward environments influence your own experiences of the ReSPECT conversation?

8. What effect does completing a ReSPECT form have on you?
    a. Earlier research on DNACPR conversations has shown that while clinicians were concerned about patients being distressed, many patients actually wanted to have these conversations; so, it has been suggested that clinicians themselves may be emotionally wary of these conversations. Do you think that's also an issue with ReSPECT?
9. Has the ReSPECT form affected how you structure your decision-making process? If so, how?
    a. Does the complexity of the form carry into your decision-making? Is it reduced into key components? If so, in which cases?
    b. Has the ReSPECT form helped you cope with uncertainty in decision-making? Why or why not?
10. Have you ever completed a ReSPECT form for a patient who lacked capacity? If so, what were the main challenges you encountered?
    a. How would you go about making a decision in the patient's best interest?
    b. With patients who do have capacity, do you face other challenges in completing the ReSPECT form?
11. Do you have any experience with ReSPECT forms completed in the community?
    a. How have you used ReSPECT forms completed in the community?
    b. How do you communicate with your patients' GPs and other community services about the ReSPECT forms you complete?
12. Has the introduction of the ReSPECT process led to improvement? Could you describe these?
13. In your opinion, what are the main flaws in the ReSPECT process?
    a. How could the process be improved?
    b. In your experience, how does the ReSPECT form compare to the DNACPR form?
14. Is there anything else you would like to say about the ReSPECT process?
